# A multiplexed magnetic tweezer with precision particle tracking and bi-directional force control

**DOI:** 10.1186/s13036-017-0091-2

**Published:** 2017-12-02

**Authors:** Keith C. Johnson, Emilie Clemmens, Hani Mahmoud, Robin Kirkpatrick, Juan C. Vizcarra, Wendy E. Thomas

**Affiliations:** 1 Mechanical Engineering, University of Washington, Seattle, USA; 2 Bioengineering, University of Washington, Seattle, USA

**Keywords:** Single molecule force spectroscopy, Magnetic tweezer, Multiplexing

## Abstract

**Background:**

In the past two decades, methods have been developed to measure the mechanical properties of single biomolecules. One of these methods, Magnetic tweezers, is amenable to aquisition of data on many single molecules simultaneously, but to take full advantage of this "multiplexing" ability, it is necessary to simultaneously incorprorate many capabilities that ahve been only demonstrated separately.

**Methods:**

Our custom built magnetic tweezer combines high multiplexing, precision bead tracking, and bi-directional force control into a flexible and stable platform for examining single molecule behavior. This was accomplished using electromagnets, which provide high temporal control of force while achieving force levels similar to permanent magnets via large paramagnetic beads.

**Results:**

Here we describe the instrument and its ability to apply 2–260 pN of force on up to 120 beads simultaneously, with a maximum spatial precision of 12 nm using a variety of bead sizes and experimental techniques. We also demonstrate a novel method for increasing the precision of force estimations on heterogeneous paramagnetic beads using a combination of density separation and bi-directional force correlation which reduces the coefficient of variation of force from 27% to 6%. We then use the instrument to examine the force dependence of uncoiling and recoiling velocity of type 1 fimbriae from *Eschericia coli* (*E. coli*) bacteria, and see similar results to previous studies.

**Conclusion:**

This platform provides a simple, effective, and flexible method for efficiently gathering single molecule force spectroscopy measurements.

## Background

Single molecule force spectroscopy (SMFS) has become a powerful tool for investigating the force dependence of biological phenomenon including, but not limited to, biological bonds [1–3],viscoelastic cell properties [4], DNA stretching [5], and motor proteins [6, 7]. However, traditional SMFS methods of atomic force microscopy, optical trap, and biomembrane force probe obtain data at slow rates, usually acquiring a single measurement at a time. Since some studies require hundreds to thousands of measurements to accurately model force dependence, such as the stochastic process of biological binding [8], gathering statistically sufficient data via traditional SMFS methods can take a prohibitively long time.

**Fig. 1:**
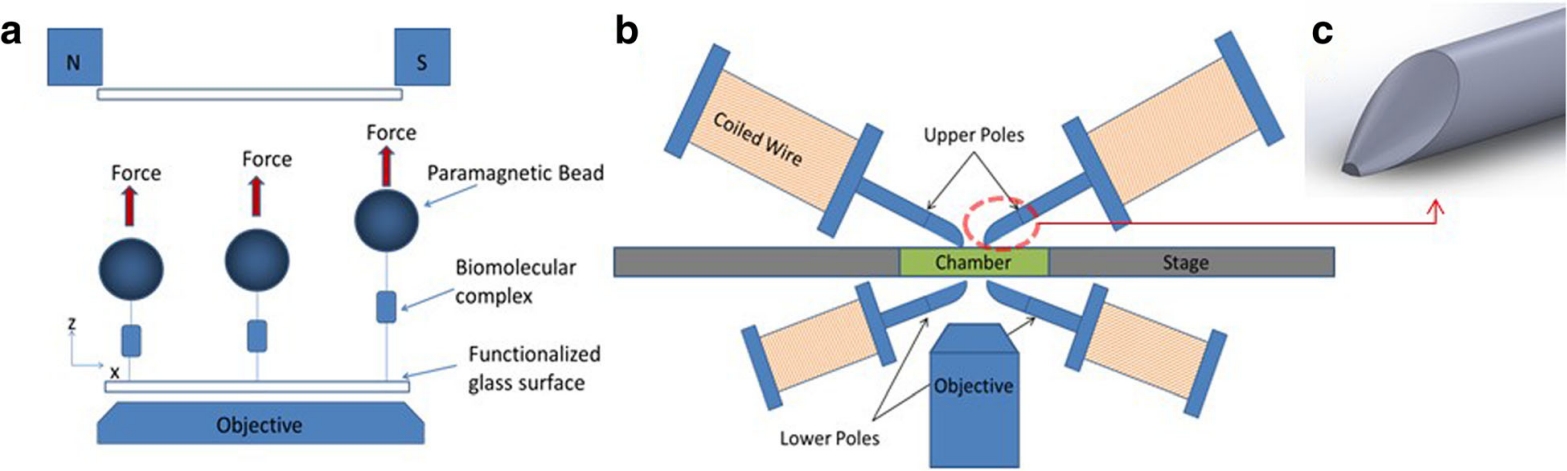
**a** An illustration of a generic MT assay. **b** An illustration of our MMTB. **c** Solid model of pole tip

The magnetic tweezer (MT) is a relatively recently developed instrument that allows the examination of hundreds of single molecule measurements simultaneously [9–11]. This ability to acquire measurements of the same phenomena simultaneously is known as multiplexing, and can significantly reduce data acquisition time when compared to other SMFS instruments. In a generic MT assay, the biomolecule or biomolecular complex has one end attached to a surface and the other end to a paramagnetic bead suspended in a chamber (Fig. 1a). The chamber can be as simple as two glass slides separated by double sided tape, containing a solution of the beads in buffer. A magnetic field gradient, generated by magnets above the chamber, pulls the beads away from the surface at constant force. The beads are viewed via a high-speed camera through the objective of an inverted microscope. By examining the position of the beads over time, force dependent properties of the biomolecular complex can be estimated. Many beads can be fit in the field of view, leading to the MT’s multiplexing capability. However, to fully take advantage of multiplexing requires the simultaneous implementation of other capabilities that have only been demonstrated separately.

The smallest detectable change in the height of a bead is referred to as the spatial resolution and determines the lower limit of biological length changes that can be detected. The spatial resolution in basic MT’s is estimated using the depth of field and is on the order of several microns [12]. However, by implementing high-precision particle tracking algorithms, the spatial resolution can be improved to ~1 nm [13–15], greatly expanding the biological questions that can be addressed [9, 11, 16–22]. The use of magnets both below and above the chamber allows beads to be pulled toward and away from the functionalized surface [12]. This bi-directional force control allows greater control of the contact time of the beads with the surface, and as will be shown, can be used to increase the precision of force estimations on the beads. To our knowledge, a MT combining the three capabilities of multiplexing, precision bead tracking, and bidirectional force control has not been demonstrated.

Here we describe a multiplexed MT with precision bead tracking and bi-directional force control (MMTB) and characterize its capabilities. Because high forces of up to 100 pN are desired for many studies, and pose unique challenges for multiplexing with electromagnets, we describe use of the MMTB to obtain this force range. To achieve these high forces, large paramagnetic beads with high magnetic content were used. These beads showed significant bead to bead force variation, which can prohibit the accurate estimation of force dependent properties. To address this challenge, we developed a novel technique for precisely determining forces on beads with heterogeneous magnetic content, which reduced the coefficient of variation of force from 27% to 6%. We then used the MMTB to examine the uncoiling and recoiling velocities of *E. coli* fimbriae under a wide range of forces. Finally, we discuss the tradeoffs when optimizing a MT for multiplexing versus spatial precision.

## Methods

### Magnetic microbeads

Information about the beads used in the experiments in this publication are shown in Table 1.

**Table 1:** Bead size, model, and manufacturer data for beads used in experiments

Bead Size (μm)	Model	Manufacturer, Location
2.8	Dynabead m-280 streptavidin	Thermo Fisher Scientific, Waltham, MA
5.3	Spherotech PM-50-10	Spherotech Inc., Lake Forest, IL
7.8	Bangs Laboratories UM4CN	Bangs Laboratories Inc., Fishers, IN
11	Spherotech CM-100-10	Spherotech Inc., Lake Forest, IL

### Chamber preparation

Chamber slides (Fisherbrand Microscope Cover Glass, 24 × 60 × 1.5, Fisher Scientific, Waltham, MA) were placed in Acetone for 3 min before being rinsed with ethanol and water. A 100 μl droplet of fimbria in 0.02% bicarbonate buffer (1.5 μg/ml) was added to center of the bottom slides and incubated for 2 h at room temperature. A 100 μl droplet of 0.2% PBS-BSA was added to the center of the top slides and incubated at room temperature for 1 h. Bottom slides were rinsed with 0.2% PBS-BSA three times. Chambers were then assembled with double sided tape and 40 μl of 0.2% PBS-BSA was injected into the chamber via a 100 μl pipet. Chambers were covered and stored overnight at 4 °C.

### Percoll centrifugation

To separate beads by density, differential centrifugation was used similarly to a method used to isolate cellular organelles of different density. Briefly, 5 ml of 1.5 M NaCl (S271–1, Fisher Chemical, Fair Lawn, NJ) was added to a Nalgene 50 ml polypropylene centrifugation tube (3119–0050, Thermo Fisher Scientific, Waltham, MA). 45 ml of a mixture of Percoll (p1644, Sigma Aldrich, St. Louis, MO) and water was added to the tube to obtain a density of 1.09 g/ml. Paramagnetic beads were washed 3 times in PBS, and added to the Percoll solution. This solution was then spun at 30,000 x g for 30 min. A thumbtack was used to create a hole in the bottom of the centrifugation tube, and the solution was drained into 1 ml aliquots. Because Percoll forms a gradient during spinning, both the percent Percoll and the spinning conditions can be adjusted to spread magnetic beads as broadly as possible for maximum separation.

### Preparation of beads for Fimbrial uncoiling experiments

In order to bind mannose-BSA to beads, 0.1 mL of 7.8 μm paramagnetic beads were washed two times in 1 mL of MES hydrate (M8250-25G, Sigma Aldrich, St. Louis, MO) with a pH of 4.5–7.5. After the second wash, the pellet was resuspended in 1 mL of MES hydrate and vortexed for 10 s. 10 mg of N-Cyclohexyl-N′-(2-morpholinoethyl) carbodiimide methyl-p-toluenesulfonate (C106402-5G, Sigma Aldrich, St. Louis, MO) was added to the beads. The beads were then vortexed for 10 s, and rotated at room temperature for 15 min. Beads were then washed two times in PBS, and resuspended in 1 mL of the same. The PBS was replaced with 1 mL of 100 mg/ml mannose-BSA (D-Mannose-BSA, NGP-1108, Dextra Laboratories, UK) in PBS and the solution was rotated at room temperature for 2 h. Beads were then washed in 1 mL of 35 mM glycine (Bio-rad Laboratories, Hercules, CA) in water with 0.2% BSA (A3059-100G, Sigma Aldrich, St. Louis, MO) and rotated at room temperature for 30 min. Beads were then washed and resuspended in 0.2% PBS-BSA and stored at 4 °C.

### Fimbria preparation

Fimbria were purified from *E. coli* using magnesium precipitation as described previously [23].

### Uncoiling model

The models in Fig. 8 were obtained via Eq. 1 [24], where *V*(*f*) is the uncoiling velocity as a function of force *f*, and using *∆L* = 5.0 nm, and *k*_*b*_ *T*= 4.114 pN-nm. The estimates of *k*_*bal*_, *f*_*bal*_, and *∆x*_*u*_ are shown in Table 2.

1 $$ V(f)=\Delta  L{k}_{bal}\left\{\mathit{\exp}\left[\frac{\left(f-{f}_{bal}\right)\Delta  {x}_u}{k_bT}\right]-\mathit{\exp}\left[\frac{\left(f-{f}_{bal}\right)\left(\Delta  {x}_u-\Delta  L\right)}{k_bT}\right]\right\} $$

**Table 2:** Fit parameters for Eq. 1, shown in Fig. 8

Parameter	Andersson [31] (Optical Trap)	Forero [24] (AFM)	Whitfield [32] (AFM)
*k*_*bal*_ (s^-1)	1.2 ± 0.9	2.2	2.29
*f*_*bal*_ (pN)	30 ± 2	22	31.3
*∆x*_*u*_ (nm)	0.59 ± .06	0.26 ± 0.1	0.461

## Results

### Description and characterization of the MMTB

#### Hardware

The MMTB has four electromagnetic poles: two above and two below a chamber containing paramagnetic beads suspended in buffer (Fig. 1b). The design and orientation of these magnets is similar to those described by Snook and Guilford [12]. Each electromagnetic pole consists of a 6.6 mm diameter Mu-metal rod (GoodFellow, Coraopolis, PA) with the tip shaped to maximize the gradient of the magnetic field [25]. This was achieved by using a taper angle of 33 degrees, with a perpendicular cut near the tip such that the cross sectional area of the flat tip of the rod is reduced by a factor of 40 when compared to its untapered area (Fig. 1c). The Mu-metal rods are encased in spools with several hundred turns of 26 AWG copper wire wound around them. The upper poles are placed very near to the top surface of the chamber, with a separation distance between the poles of ~1 mm. The lower poles, due to spatial constraints below the chamber, are spaced ~7 mm apart and ~3 mm below the chamber. This reduces the magnetic field that pulls beads down, but suffices.

By applying a voltage potential across the coils using two identical power supplies (1697, BK Precision, Yorba Linda, CA), a magnetic field gradient acts to pull the beads in the upward (upper magnet) or downward (lower magnet) direction. In practice, the lower magnet is first used to pull the beads to the bottom, functionalized surface. The current to the lower magnet is then turned off and the current to the upper magnet turned on, and the beads are pulled away from the bottom surface at a force controlled by the magnetic field. This cycle of pulling the beads toward and away from the bottom surface is known as a “pull”. A high speed camera (GT1910, Allied Vision, Exton, PA) is mounted to an inverted microscope (Eclipse TI-E, Nikon, Melville, NY) and used to acquire images of the beads at rates of up to 100 Hz, using either a 0.45 NA 20× or 0.55 NA 40× objective. The use of objectives with longer working distances and low NA’s is due to spatial constraints below the chamber caused by the lower magnet. With the 20× objective, 40 7.8 μm diameter beads or 120 2.8 μm diameter beads can fit in the field of view (528 × 297 μm), with enough space between beads to allow bead tracking i.e. diffraction patterns do not overlap (see Bead Tracking). Multiplexing can be further increased using non-random tether techniques [9].

A custom Labview program (National Instruments Corporation, Austin, TX) is used to provide synchronous control of the microscope, power supplies, and camera. The Labview program allows the camera and power supply settings to be adjusted in real time, or a time-based script can be used to ensure repeatability between pulls. This flexibility is beneficial when measuring bond lifetimes: one may use a high frame rate to examine the rupture of short-lived bonds, and then switch to a lower frame rate to examine the rupture of long-lived bonds, thus avoiding collecting superfluous amounts of data. Using the Labview program, we found that it takes ~40 ms to switch from one current level to another (data not shown), similar to the findings by Snook and Guilford [12].

#### Bead tracking

**Fig. 2:**
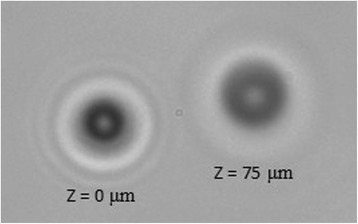
Example diffraction patterns of beads on the bottom surface of the chamber (z = 0 μm) and at the top of the chamber (z = 75 μm)

Experimental images are analyzed using a custom Matlab script (MathWorks, Natick, MA) that implements a variation of previously published tracking algorithms [13, 14]. In short, as the spherical paramagnetic beads travel in the z-direction (along the axis of the objective), the diffraction pattern of the beads change as they move in or out of focus (Fig. 2). This change in diffraction can be used to estimate the z-position (height) of the beads. This process involves first acquiring images of a representative bead at different axial positions by moving the objective small axial steps while the beads are on the bottom surface. These calibration images can then be compared to each experimental image, when the beads are moving and the objective is stationary, and used to estimate the z-position of the beads in each experimental image. This method requires a scalar correction factor to account for the air/water refractive index mismatch. Also, beads should be spherical in shape and have a uniform size.

To estimate the tracking accuracy of the MMTB over long distances, we tracked several beads under force as the beads traversed the axial distance of the chamber(~78 μm) under 0.6 amps of current. We then compared this to the chamber height, as measured by focusing on beads that were stuck to the top and bottom of the chamber and noting the microscope objective position. The difference in objective position was then multiplied by 1.33 to account for the refractive index mismatch. The tracked bead displacement was within a few percent of the chamber height with an error of 2.9 ± 0.5% (SEM) for 7.8 μm beads. This demonstrates that our system is capable of accurately tracking beads over long distances, an important ability when estimating the force on beads (described in Force Calibration).

**Fig. 3:**
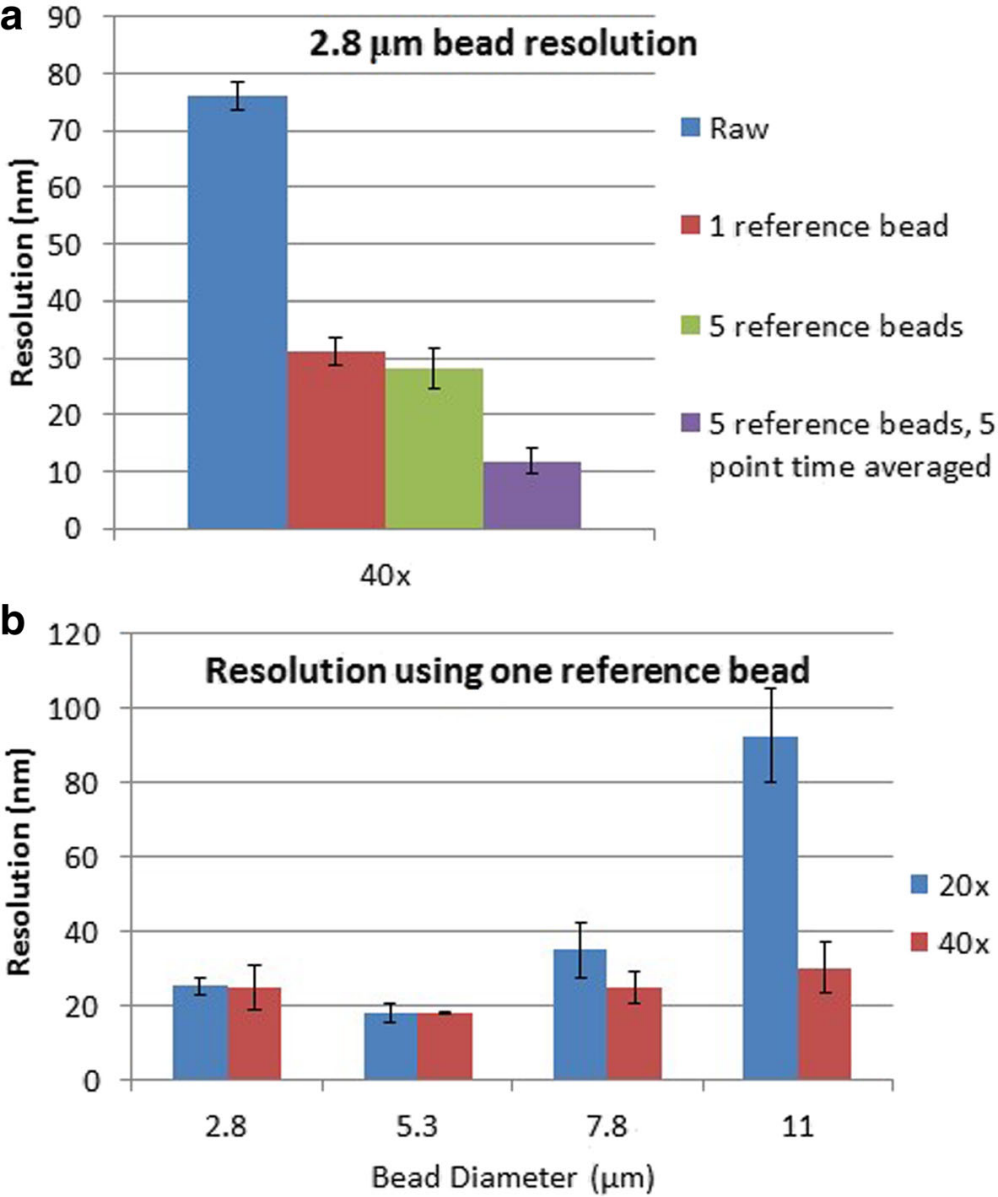
**a** Resolution of 2.8 μm beads for different conditions using a 0.55 NA 40× objective. **b** Resolution using different sized beads when subtracting one reference bead using a 0.45 NA 20× objective and a 0.55 NA 40× objective. All error bars represent the standard error of the mean (SEM) of two separate data sets of 3–9 measurements obtained on different days

To test the spatial resolution, we non-specifically bound beads to the surface, and determined their z-positions over time under no force (0 amps of current). The resolution was computed as the standard deviation of the bead displacement. We determined this resolution at 40× magnification under various conditions for 2.8 μm diameter beads (Fig. 3a). Examining raw data, the resolution was ~75 nm, which may primarily reflect stage vibration. By subtracting the displacement of another bead in the field of view, or reference bead, movement of the stage was accounted for and the resolution was improved to 30 nm. Subtracting the average displacement of five reference beads showed no or a small improvement over the single reference bead. Using a combination of five reference beads and a five-point rolling time average of position, resulted in a resolution of 12 nm. However, this rolling time average sacrifices temporal resolution, and therefore may not be suited for some studies. This data shows that stage vibration is a major factor for spatial resolution, and that precision cannot be optimized without sacrificing temporal resolution.

To examine the effect of objective properties and bead size on spatial resolution we determined the spatial resolution for beads of different sizes using a 0.45 NA 20× and a 0.55 NA 40× objective while using a single reference bead (Fig. 3b). At 20×, the resolution was similar for the 2.8–7.8 μm beads but increased dramatically for the 11 μm beads. At 40×, the resolution was a relatively constant 18–30 nm for all bead sizes. Considering the twofold higher magnification objective results in a four-fold reduced field of view and throughput, the small improvement in resolution may be unwarranted for most experiments. Also, since the resolution of beads ranging from 2.8–7.8 μm was similar, the choice of bead size should be based on the desired force range and level of multiplexing: larger beads can achieve higher forces, but fewer can be tracked simultaneously.

#### Force calibration

The force on the beads was estimated by determining the z-position of the beads as a function of time and then applying a modified version of Stokes’ law,

2 $$ F=6\pi \mu rv\lambda $$

where *F* is the drag force on the bead, *μ* is the dynamic viscosity of the fluid, *r* is the radius of the bead, *v* is the velocity of the bead, and *λ* is a correction factor due to the chamber surface effects [26] that depends on the relative values of the bead radius and the distances to the near (b) and far (c) surfaces. When the bead is far from the nearest surface (b/*r* > 15), or one surface is much closer (b/(b + c) < 0.2), the effect of the distal surface is insignificant [27], so lambda can be estimated using the empirical approximation for a single surface [26]:

3 $$ \lambda =1+1.08\ \left(\frac{r}{b}\right)+1.4{\left(\frac{r}{b}\right)}^2 $$

Considering that our average chamber height was approximately 78 μm, this estimation of lambda was used for estimating forces on 2.8 μm and 5.3 μm beads, where the position of the bead was assumed to be in the middle of the chamber (b = 39 μm). Because bead velocities were typically measured in the middle third of the chamber, we estimate that this approximation yielded an error in force of less than 5%. For the larger 7.8 μm and 11 μm beads, b/r was less than <15, and therefore the second surface had an effect on the bead velocity. In this case, *λ* was estimated using the tables and figures created by Ganatos, Pfeffer, and Weinbaum [27]. Estimations of *λ* in the middle third of the chamber ranged from 1.04 for 2.8 μm beads to 1.35 for 11 μm beads. It should be noted that taller chambers would minimize the need to compensate for surface effects when calibrating force even with larger beads.

To assess the magnetic field gradient variation in the chamber along the z-dimension, we examined the velocity of beads at two different heights of the chamber. Because the velocity of beads near the chamber walls are highly nonlinear due to the effect of the chamber walls, we examined the change in bead velocities at positions in the chamber where the velocities (and thus forces) were more stable: when the beads were at z-positions 25% and 75% the height of the chamber. Examining the velocity of fifteen 7.8 um beads measured on two separate days, we found that the velocity of the beads at a z-position of 75% of the chamber height is 1.11 ± .02 (SEM) times that of the beads at 25% of the chamber height. This increase is unsurprising as the beads are slightly closer to the magnets at the 75% height. Since this change in height is the same as when beads are on the surface compared to when they are halfway across the chamber (where the force is calibrated), we expect the forces measured at the halfway point to be within 11% of the forces at the surface. This potential 11% force error is very similar to the force accuracy of other SMFS instruments [28].

**Fig. 4:**
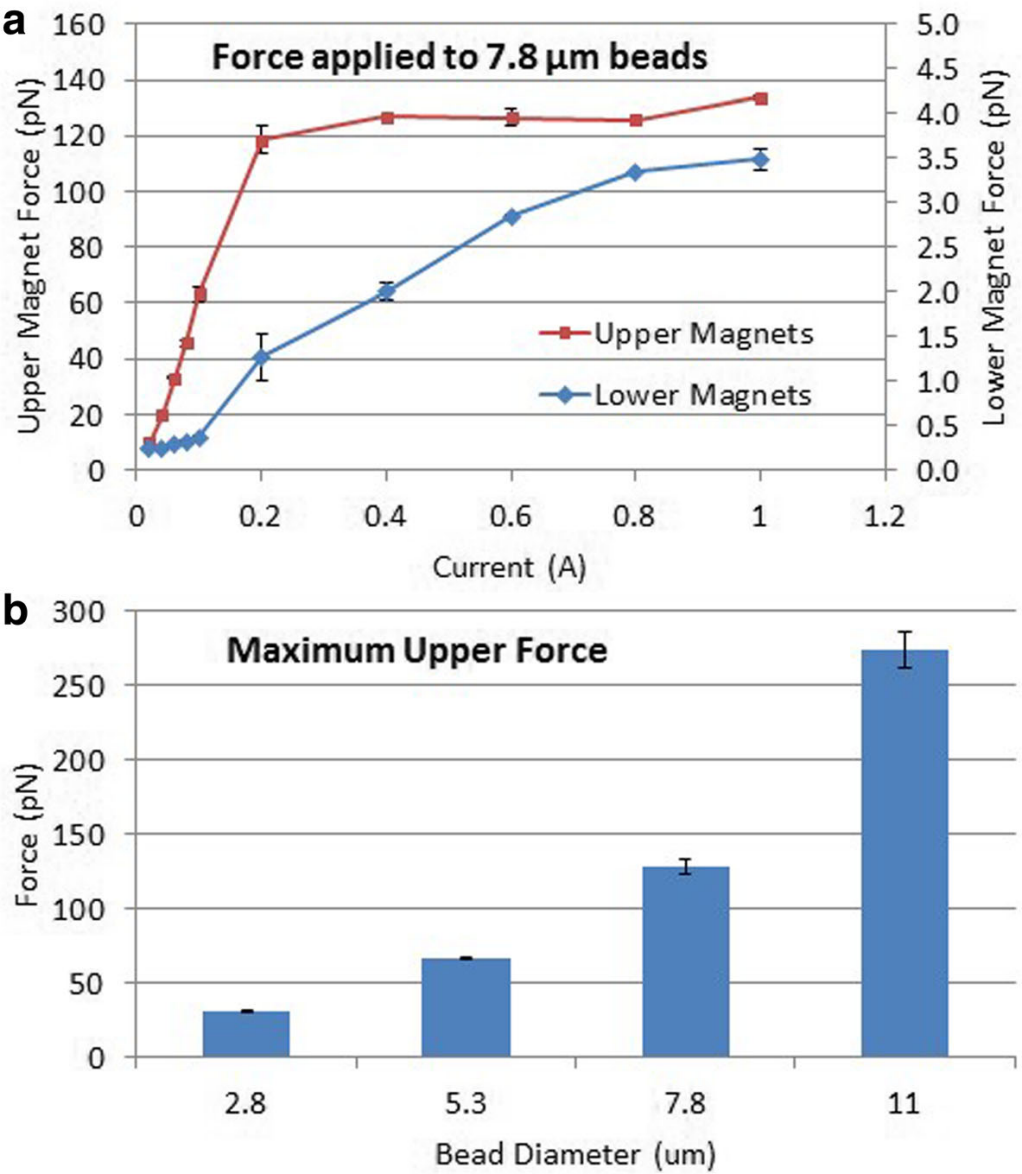
**a** Force on 7.8 μm diameter beads for different levels of current for both the upper and lower magnet. **b** Maximum upward force at 0.6 amps for beads of different sizes. All error bars represent the standard error of the mean (SEM) of two separate data sets of 5–10 beads obtained on different days

We determined the force on 7.8 μm beads at different currents, for both the upper and lower magnet (Fig. 4a). For the upper magnet, the force increased linearly with current to 120 pN at 0.2 amps, and any further increase in current resulted in only minor force gains. That is, the MMTB provided a linear relationship between force and current within the entire working range. For the lower magnet, the linear force region extended to ~3.5 pN at 1 amp before plateauing. The difference in current needed to saturate the magnetic field for the lower versus upper magnet is because the lower magnet has a different pole shape and number of wire coils than the upper magnet due to spatial constraints below the chamber. To examine the maximum force for different sized beads, we measured the force at 0.6 amps for 2.8–11 μm diameter beads (Fig. 4b). This resulted in forces ranging from 32 pN with 2.8 μm beads, to 260 pN for 11 μm beads.

The maximum downward force of 3.5 pN at 1 amps for the 7.8 μm beads allows an estimate of the contact time resolution of the beads with the bottom surface using Eq. 5,

4 $$ {C}_{95}={h}^{\ast}\left[\frac{6\pi \mu r}{F_{down}-{2}^{\ast }{COV}^{\ast }{F}_{down}}-\frac{6\pi \mu r}{F_{down}+{2}^{\ast }{COV}^{\ast }{F}_{down}}\right] $$

where *C*_95_ represents the difference in contact time of 95% of the bead population, *F*_*down*_ is the force applied with the lower magnet, COV is the coefficient of variation in force for the beads, and *h* is the height of the chamber. Using our maximum downward force of 3.5 pN and *COV* of 0.14 (see Force Variation), we estimate that 95% of beads will contact the surface within 1 s of each other. Eq. 5 shows that the contact time resolution can be improved by decreasing *h* or *COV*, or by increasing *F*_*down*_.

Similarly, the combination of the upward force and spatial resolution controls the bond temporal resolution, or the shortest bond lifetime that can be estimated.

5 $$ {\beta}_B\approx \frac{6\pi \mu r\eta}{F}, $$

where *β*_*B*_ is the bond temporal resolution, and *η* is the spatial resolution. For the 7.8 μm beads, the spatial resolution without reference beads or time averaging was about 75 nm. At the lowest force of 10 pN, the bond temporal resolution is estimated to be 0.5 ms using Eq. 6. At 100 pN this value decreases to 0.05 ms. Typically, a camera frame rate of 50 Hz is used, and thus we conclude the bond temporal resolution is frame rate limited to 20 ms. A faster frame rate is achievable by reducing the pixels per field of view, so higher bond temporal resolution is possible if some multiplexing may be sacrificed. In contrast, without bead tracking the spatial resolution is estimated as the depth of field (2.78 μm) and the bond temporal resolution at 10 pN becomes 20 ms and is therefore limited by tracking, not image acquisition.

Expanding on this force data, the smallest upward force that can be applied using the MMTB is about 2 pN with 2.8 μm beads (data not shown), and is limited by the lowest applicable current of our power supply of 0.02 Amps. This force is about five times smaller than the smallest applicable force using an atomic force microscope [29], and could be further decreased by using a more versatile power supply or increasing the space between the magnetic pole tips. Similarly, the largest force that can be applied with the MMTB is about 260 pN with 11 μm beads at 0.6 Amps (Fig. 4b). This force is ~2.5 fold higher than the maximum applicable force with an optical trap [29]. This 130-fold force range with commercially available beads encompasses the forces usually seen when investigating biological phenomena [1–3, 5]. Furthermore, the linear relationship between force and current over the working range of forces in the MMTB (see Fig. 4a), combined with the ability to program any desired change in current, provides time-dependent force control without the need for a feedback loop. Together, the biologically relevant force range and simple mechanism for manipulating force makes the MMTB a versatile instrument for acquiring constant force measurements.

#### Force variation

**Fig. 5:**
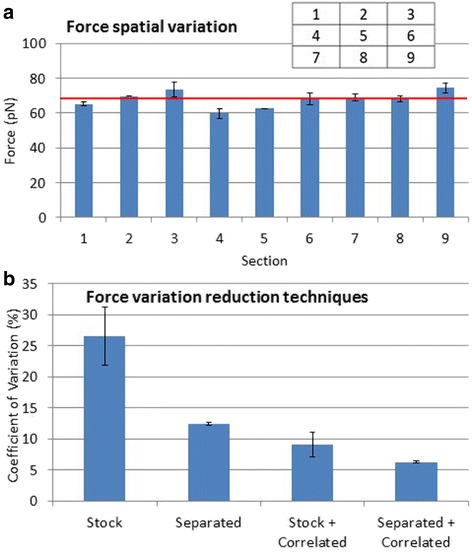
**a** Force applied to 7.8 μm diameter beads in different 176 × 99 μm sections of the field of view (inset) with average force of all sections shown in red. **b** Coefficient of Variation (COV) in force using various techniques. All error bars represent the standard error of the mean (SEM) of two separate data sets of 8–20 beads obtained on different days

To assess the variation in force across the field of view, we parsed the field of view into nine sections of 176 × 99 μm per section (Fig. 5a, inset), and determined the average force on beads within each section (Fig. 5a) at 0.1 amps. The average force over all the sections was 68 pN, while section 4 showed the smallest force of 60 pN and section 9 showed the largest force with 74 pN. A two-tailed t-test using unequal variances found that no section was statistically different from the average when using a critical *p*-value of .00625, in accordance with Dunn [30]. This range of forces is likely due to a magnetic field gradient that was not perfectly homogenous across the field of view. Changing the magnetic pole tip shape or increasing the space between the magnetic pole tips can help create a more homogenous magnetic field gradient.

To test the bead to bead force variation, we determined the coefficient of variation (COV) in force of 7.8 μm beads at 0.1 amps. This resulted in a COV of 27% for “Stock” beads: beads directly from the manufacturer’s container (Fig. 5b, Stock). We hypothesized that this high value was due to inconsistent amounts of magnetic material across the bead population, which would manifest in different bead densities. To test this hypothesis, we separated beads by density using Percoll (p1644, Sigma Aldrich, St. Louis, MO) to create a centrifugal density gradient. We then extracted a small portion of beads from the middle of the density gradient and examined the COV. The separated beads had a 12% COV (Fig. 5b, Separated), less than half the COV of the stock beads.

To further increase the precision of the bead force estimations, we developed a method that correlated the downward force (toward the bottom surface with the lower magnet) and the upward force (away from the bottom surface with the upper magnet). Due to the difference in the amount of magnetic material across the bead population, there was a correlation between the downward force and upward force of individual beads, which could be used to increase the precision of the upward force estimations of adhered beads. This process began with a calibration step, where the downward and upward force on tens of beads was acquired. This data was used to fit parameters *m* and *y* in Eq. 7,

6 $$ {F}_{upper,\alpha }={m}^{\ast }{F}_{lower,\alpha }+y $$

where *F*_*upper*, *α*_ is the estimated force on the bead using the upper magnet at current *α*, and *F*_*lower*, *α*_ is the measured force on the bead using the lower magnet at current *α*. Proceeding pulls, where the beads were initially pulled down to the bottom surface, were used to estimate the upward force on beads that had adhered to the bottom surface using the initial downward force, and parameters *m* and *b* in Eq. 7. An estimate of the precision in upward bead force was determined by comparing the linear model (Eq. 7) to the calibration force measurements, and calculating the standard deviation of the error. This correlation method resulted in a COV of 9% for stock beads, and a COV of 6% for beads that had been separated (Fig. 5b). Thus, by combining the separation and correlation methods, the COV was reduced by 78% when compared to stock beads.

### Application of MMTB for single molecule force measurements

**Fig. 6:**
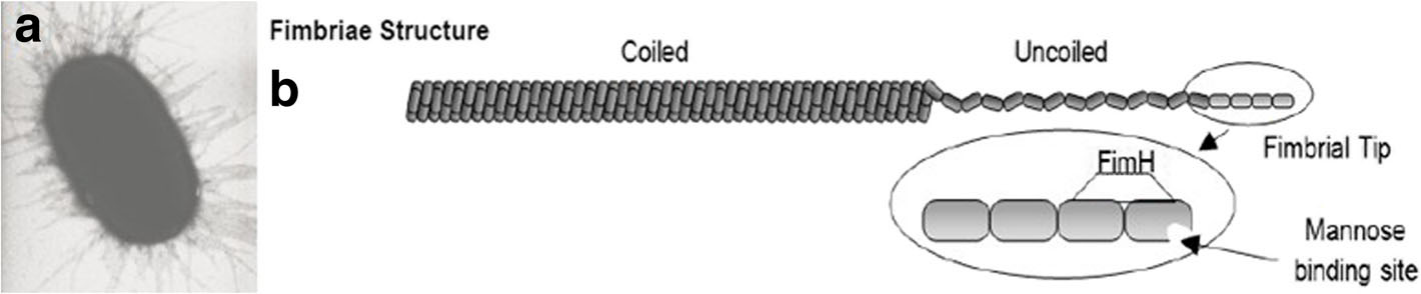
**a** Electron micrograph of *Eschericia coli* (*E. coli*). Figure altered from Thomas [49] **b** Illustration of fimbria structure. Figure taken directly from Whitfield and Thomas [33]

To test the MMTB for use in making biological measurements, we measured the uncoiling and recoiling velocity of type 1 fimbriae from *E. coli* (Fig. 6a). We chose this system because it demonstrates the utility of many of the characteristics of the MMTB, including spatial precision, multiplexing, and bi-directional force control. Type 1 fimbriae have also been studied previously [24, 31, 32] and thus a comparison can be made to measurements taken with more traditional SMFS methods. Briefly, fimbriae are approximately 1 μm appendages found on the surface of *E. coli*. On the tip of each fimbria is the protein FimH, which binds strongly to mannose under force [3]. The fimbria itself is composed of many helical coils of the subunit FimA, and when enough force is applied to the fimbria, the FimA subunits can uncoil sequentially (Fig. 6b) [24]. If the force on an uncoiled fimbria is reduced sufficiently, recoiling will occur at high velocity. Fimbrial mechanics are a key element in *E. coli’s* ability to adhere to mannosylated surfaces under fluid shear stress [33, 34].

**Fig. 7:**
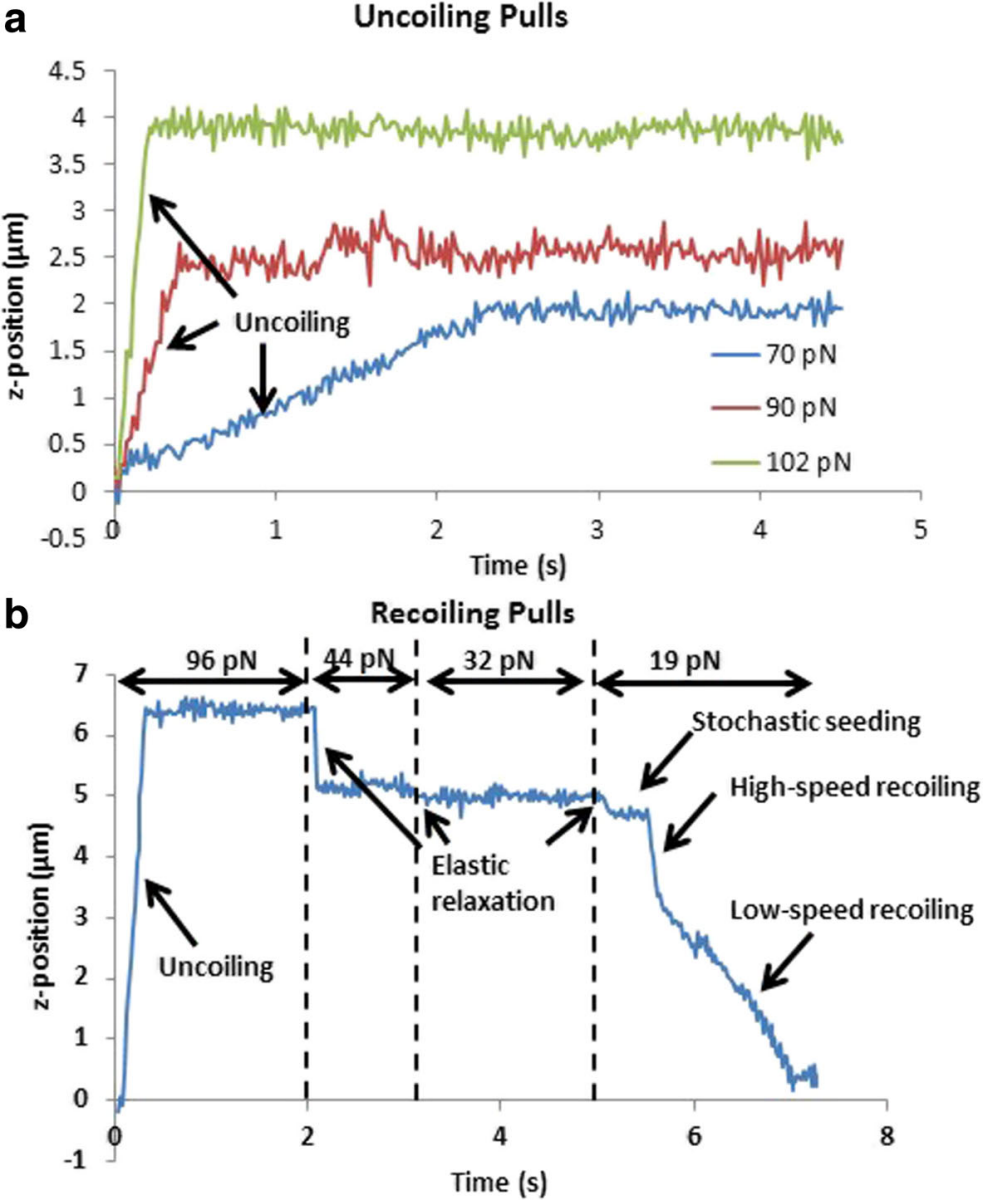
**a** Position data for three fimbriae that were pulled at different forces. **b** Position data for one fimbria during a recoiling pull

In our experiment, 7.8 μm carboxyl coated paramagnetic beads were separated by density using Percoll centrifugation, and then covalently linked to Mannose-BSA while fimbriae were non-specifically adsorbed to the glass surface on the bottom of the chamber. Beads, initially near the top surface of the chamber, were pulled down to the bottom surface using the lower magnet and after approximately 1 s of contact with the surface, pulled away from the surface using the upper magnet. For pulls examining the uncoiling velocity, forces ranging from 46 to 127 pN were applied to the beads and held for 5 s allowing the fimbriae to elongate (Fig. 7a). For pulls examining recoiling velocity, after an initial upward force of ~100 pN was applied for 2 s to fully extend the fimbria, the force was reduced in a stepwise manner to about 45, 32, and 20 pN respectively (Fig. 7b). In this way, multiple recoiling force measurements were made with a single pull. The forces on beads that did not adhere to the surface, and thus could be viewed approaching and leaving the surface, were used to create a correlation model of upward force as a function of downward force for different current levels. These models were used to estimate the force on the attached beads. Two data sets from multiple days of experimentation were acquired.

To demonstrate specific adhesion, beads that did not have covalently bound Mannose-BSA were pulled from the fimbria-coated surface at either 60 or 100 pN. These negative control pulls had less than 1% (SEM = 0.2%) adhesion on average, while the average for beads with Mannose-BSA had 23% (SEM = 5%) of beads adhered to the surface. This demonstrates that the vast majority of adhesive events were bound specifically to the fimbrial tips. Based on the net 22% adhesion rate, a Poisson distribution predicts that 12% of attached beads formed multiple attachments [35].

To ensure that measured velocities were due to fimbrial uncoiling and not reorientation of long fimbriae, we used atomic force microscope imaging to measure the lengths of the fimbriae, which were found to have an average native length of 0.43 ± 0.15 μm with no fimbriae longer than 0.8 μm (*N* = 39 samples). We then disregarded any measurements that had bead displacements of less than 1.5 μm during the 5 s uncoiling pulls, or during the 2 s 100 pN force for the recoiling pulls. This eliminated any measurements in which fimbriae changed orientation to an upright position without uncoiling, since it is very unlikely that any single fimbria had a length greater than 1.5 um in the native state. Because uncoiling typically extends fimbria to ten times their native length [24], many uncoiled fimbria met this criteria. We also required at least a 225 nm bead displacement during the fimbrial recoiling velocity measurements, which ensured that all measurements were beyond position noise (~75 nm for this experiment).

A key part of the analysis was correctly interpreting the different parts of the displacement curve for recoiling pulls (Fig. 7b). The beads were initially pulled away from the bottom surface at high force (~100 pN) to fully extend the fimbria. When the force was lowered, there was an instantaneous relaxation due to the nonlinear elasticity of the fimbria [32]. After the force was reduced to its lowest level (~20 pN), there was sometimes a delay before the high-speed retraction began. An analysis of these delays found an average delay time of 0.15 ± 0.27 s, with most of the fimbriae beginning to recoil instantaneously after the force drop, while ~15% showed delay times of a few tenths of a second or more. We interpreted this as the time it takes for the formation of a nucleation kernel, which has been previously suggested [31]. We found that there were often two speeds of retraction, with the higher velocity always being the initial velocity. This has also been observed previously with type 1 pili, and is hypothesized to be two different methods of recoiling [31]. We used the initial high-speed velocity for the analysis since it always occurred, whereas the low-speed recoiling was not always observed. Lastly, because the beads moved at large velocities during uncoiling and recoiling, the drag force on the beads needed to be taken into account to accurately determine the total force. During uncoiling pulls, the drag force acts in the opposite direction of the force from the magnet.. Thus, to determine the uncoiling force, the drag force should be subtracted from the magnet force,

7 $$ {F}_{uncoiling}={F}_{magnet}-{F}_{drag} $$

Similarly, for recoiling pulls, the drag force should be added to the magnet force to determine the recoiling force,

8 $$ {F}_{recoiling}={F}_{magnet}+{F}_{drag} $$

The drag force was determined using Eq. 3, with lambda estimated from Eq. 4.

**Fig. 8:**
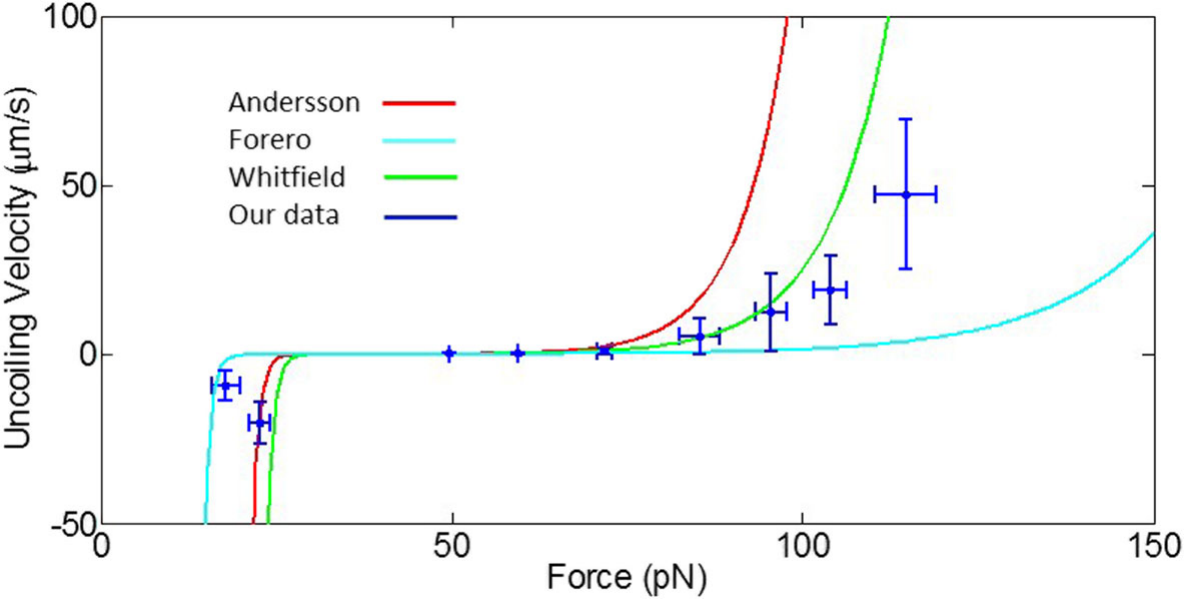
Uncoiling and recoiling velocities as a function of force. Solid lines represent the fit using parameter values in Table 2 of previously published data by Andersson [31], Forero [24], and Whitfield [32]. The combination of our two data sets binned into 10 pN bins is shown as blue crosses, where error bars represent the standard deviation of measurements within each bin

Figure 8 shows the uncoiling (velocities >0 μm/s) and recoiling (velocities <0 μm/s) velocities and their corresponding forces for both of our data sets. To compare our data to previous publications, we also plotted the models of Andersson [31], Forero [24], and Whitfield [32], all of whom did similar studies using traditional SMFS instruments. These models are shown in Fig. 8 as solid lines. These previous measurements vary considerably, suggesting differences in bacterial strains, buffer conditions, or instrument calibration. Our new measurements are most consistent with the measurements of Whitfield et al., which were performed in our lab with the same strains and buffer conditions, but with a different instrument. There is about a two-fold difference between our data and the Whitfield data in the high force regime in terms of the *velocity* for a given force. This is expected, because a velocity is a type of rate, and two-fold differences in rate constants measured in kinetic experiments are routine. However, there is only a small difference in *forces* needed to obtain the same velocity. The difference in force at the largest forces we tested (~115 pN) is about 10 pN, or <10% of the force applied. This discrepancy can thus be explained by the 10% error in force accuracy commonly seen using SMFS instruments [28]. More details on the model fit shown in Fig. 8 can be found in Methods.

## Discussion and conclusions

### Advantages of MMTB

Our MMTB with its unique combination of multiplexing, precision bead tracking, and bi-directional force control was able to efficiently gather single-molecule uncoiling and recoiling velocities over a wide range of forces. Without any of the three aforementioned capabilities, this study would have been significantly more difficult.

The multiplexing ability of the MMTB decreased the experimental time needed to acquire the data. In this case, the decrease was not large over traditional methods. Most of our pulls lasted only 6 s for the actual pull plus 60 s to replace the beads in the chamber, and provided an average of more than 30 measurements per pull, or 0.45 measurements per second. To run a similar experiment with traditional SMFS instruments, there would only be 1 measurement for every 6 s pull, or 0.17 measurements per second. We therefore estimate the MMTB acquired data ~2.7 times faster than traditional SMFS instruments for this experiment. The major limitation on efficiency in this experimental was that detached beads aggregate under the influence of the magnetic field, so beads cannot be reused for multiple pulls, requiring the minute-long bead replacement after each pull cycle. For long pulls where efficiency is most critical, this additional time is well worth it to provide multiplexing. However, for short pulls of less than 1 s, high efficiency would require improvements such as creation of regular arrays with microcontact printing [9], a microfluidic device for rapid bead replacement, and/or an experimental design that eliminates complete detachment of beads from surface.

Multiplexed SMFS systems have been demonstrated with other MT’s [11, 12], and alternative methods including acoustic force spectroscopy [36], centrifugal force spectroscopy [37], nanophotonic traps [38], optoelectronic tweezers [39], AFM cantilever arrays [40], optical tweezer arrays [41], and DNA curtains [42]. However most of these systems lack key attributes of the MMTB including the ability to change force quickly [37], precision bead tracking [12], bidirectional force control (and thus the use of force correlation) [11], and application of large forces [39]. The multiplexing arrays of optical tweezers or AFM cantilevers do not allow independent position control or force control through feedback loops, so do not apply uniform force conditions to all elements of the array [40, 41]. Other methods have issues with local heating [38, 43], or use high optical intensities that can damage biological specimens [39, 44]. MT’s therefore provide the most flexible multiplexed platform for biological measurements at this time.

For MT’s without precision bead tracking, the spatial resolution is estimated as the depth of field and is on the order of several microns [12]. Since the fimbriae typically uncoiled to lengths of <8 μm, the uncoiling velocities would have been inaccurate or even undetectable without tracking. However, even without using reference beads or time averaging, our spatial resolution of 75 nm was sufficient to accurately determine the uncoiling velocities in this study.

The ability to quickly change the bead force was imperative in estimating recoiling velocities. Because the recoiling velocities were quite rapid, there was a limited time at which the force-dependent velocity could be measured. Since we wanted to measure the dependence of this recoiling velocity on force, the force on the beads had to be set quite quickly, otherwise the fimbria would have completely recoiled before the new force level was attained. This was achievable with electromagnets where a new steady-state force was reached in ~40 ms, but would be more difficult with permanent magnets because a precise and fast shift in position would be required.

Finally, our novel force correlation method, which required the use of the lower magnet, allowed force estimations of tethered beads with greatly improved precision. An alternative method for estimating bead forces uses the Brownian motion of the tethered bead and requires a known tether length and a significant amount of bead position data [45]. This method would have been difficult to implement for the recoiling pulls since the fimbria length was dependent on the bead force, and the bead force was changed every 1–2 s resulting in few data points at each force (Fig. 7b). Our method can be used for virtually any type of experimental design with the MT. Use of the lower magnet to bring the beads into contract with the reactive surface also provides higher temporal control of contact time than does the use of gravitational settling.

### Optimization of MMTB for different purposes

Here we specifically designed and optimized the MMTB for efficiently gathering mechanical measurements of single molecules or molecular complexes. This necessitated both multiplexing and precision bead tracking, but prevents either of these capabilities from being maximized. However, others in the field may have need of instruments that emphasize other applications of SMFS, and we offer the following guidance for those considering building their own MT.

To maximize the multiplexing ability of the MT, as many beads as possible should be fit into the field of view. This can be achieved by using a camera with a large field of view in combination with a low magnification objective and small beads. Such a setup does come with drawbacks. First, cameras with large field of views often have low frame rates. This frame rate reduction combined with the low magnification objective decreases the maximum spatial precision [10]. Also, obtaining a uniform magnetic field gradient over a large area requires either more space between the magnetic poles or a less tapered pole tip (for electromagnets) [25], which reduces the maximum bead force that can be achieved. This is compounded by the desire to use small beads, which typically have a lower applied force than larger beads, due to the smaller amount of magnetic content.

Conversely, maximum spatial precision requires a high magnification objective and a camera with a high frame rate and small pixel area. Such cameras tend to have smaller fields of view, which together with the higher magnification reduces the multiplexing factor. For very high speed cameras, a stronger light source may be required for proper illumination, of which superluminescent diodes are an option [46]. Due to the smaller field of view, a smaller area of uniform magnetic field gradient will suffice, allowing the magnetic poles to be placed closer together, or a more tapered electromagnetic pole tip to be used. These magnetic pole setups can obtain larger maximum bead forces when compared to the multiplexing setup. Finally, since current bead tracking algorithms assume a spherical shaped particle, beads with a very uniform shape will be required to maximize spatial precision. With such a setup, spatial precisions of 0.1 nm at 100 Hz have been demonstrated [46].

The preceding paragraphs have shown the spectrum of capabilities of the MT: maximizing multiplexing and throughput on one end, and maximizing spatial precision and bead force on the other. Our MMTB is a hybrid of these two extremes: capable of enough spatial precision to detect many biological phenomena [20, 31, 47, 48], while multiplexing to a degree that dramatically increases the throughput of many SMFS experiments. This increased throughput, the biggest advantage of the MT over other SMFS instruments, increases the scope and complexity of questions that can be addressed using SMFS. We hope that this article is a useful guide for others in the field that may be interested in developing or optimizing their own MT.
